# The MADS-box genes expressed in the inflorescence of *Orchis italica* (Orchidaceae)

**DOI:** 10.1371/journal.pone.0213185

**Published:** 2019-03-01

**Authors:** Maria Carmen Valoroso, Maria Concetta Censullo, Serena Aceto

**Affiliations:** Department of Biology, University of Naples Federico II, Napoli, Italy; National Taiwan University, TAIWAN

## Abstract

The Orchidaceae family, which is one of the most species-rich flowering plant families, includes species with highly diversified and specialized flower shapes. The aim of this study was to analyze the MADS-box genes expressed in the inflorescence of *Orchis italica*, a wild Mediterranean orchid species. MADS-box proteins are transcription factors involved in various plant biological processes, including flower development. In the floral tissues of *O*. *italica*, 29 MADS-box genes are expressed that are classified as both class I and II. Class I MADS-box genes include one Mβ-type gene, thereby confirming the presence of this type of MADS-box genes in orchids. The class II MIKC* gene is highly expressed in the column, which is consistent with the conserved function of the MIKC* genes in gametophyte development. In addition, homologs of the *SOC*, *SVP*, *ANR1*, *AGL12* and *OsMADS32* genes are expressed. Compared with previous knowledge on class II MIKC^C^ genes of *O*. *italica* involved in the ABCDE model of flower development, the number of class B and D genes has been confirmed. In addition, 4 class A (*AP1/FUL)* transcripts, 2 class E (*SEP)* transcripts, 2 new class C (*AG)* transcripts and 1 new *AGL6* transcript have been identified. Within the *AP1/FUL* genes, the sequence divergence, relaxation of purifying selection and expression profiles suggest a possible functional diversification within these orchid genes. The detection of only two *SEP* transcripts in *O*. *italica*, in contrast with the 4 genes found in other orchids, suggests that only two *SEP* genes could be present in the subfamily Orchidoideae. The expression pattern of the MIKC^C^ genes of *O*. *italica* indicates that low levels at the boundary of the domain of a given MADS-box gene can overlap with the expression of genes belonging to a different functional A-E class in the adjacent domain, thereby following a “fading borders” model.

## Introduction

Among the flowering plants, the monocot family Orchidaceae is one of the most species-rich and widespread; this family has adapted to different habitats and exhibits highly specialized reproductive strategies [1]. The Orchidaceae family includes five subfamilies (Apostasioideae, Cypripedioideae, Vanilloideae, Epidendroideae and Orchidoideae) and numerous tribes and subtribes [2]. One of the most attractive orchid structures is the flower that assumes an enormous variety of shapes and colors among the species although it has a generally conserved structural organization. Three outer tepals, two lateral inner tepals and an inner median tepal (labellum or lip) define a zygomorphic perianth. Male and female reproductive tissues are fused to form the gynostemium or column, and pollen grains are located at the top of this structure. The ovary is located at the base of the column, and its maturation is triggered by pollination [3].

MADS-box genes play a crucial role in the evolution of flower architecture. This family of transcription factors is divided into two lineages, types I and II, that differ in genomic organization, developmental roles, evolutionary rate and level of functional redundancy [4].

Type I MADS-box proteins contain the MADS domain and are divided in Mα, Mβ and Mγ based on sequence divergence at the C-terminus [4]. These proteins are involved in seed, embryo and female gametophyte development [5]. Type II MADS-box proteins are the most studied lineage of MADS-box genes given their involvement in different plant developmental processes, including flower formation. These transcription factors are characterized by four domains, including three variably conserved domains and one variable domain, that form the MIKC structure. The highly conserved MADS domain has DNA-binding activity and recognizes the CArG-box motifs in the target genes [6]. The less conserved I and K domains are involved in protein-protein interactions and the formation of protein complexes [7, 8]. The variable C domain has a role in the formation of protein complexes and confers specific functional activity [8, 9].

A duplication event involving the 5’ region of the exon encoding for the K domain followed by neofunctionalization gave rise to two classes of MIKC-type genes: MIKC^C^ and MIKC* [10]. The MIKC* genes are involved in male gametophyte development [11], whereas the MIKC^C^, the most studied MIKC genes, play different roles in various processes of plant growth and the establishment and maintenance of floral organs [12].

The MIKC^C^ genes involved in flower organ formation are divided into five functional classes (from A to E), and their activity is described by the ABCDE model of flower development. The MADS-box genes involved in this model form homo- and heterodimers (floral quartets) that regulate specific expression programs in different floral whorls [13].

The ABCDE floral quartet model is applicable to different plant species, where it is well conserved [14–17]; however, analyses of non-model species have revealed differences related to the different structures of the flower [18]. For example, in orchids, the class B MADS-box genes exhibit an expression profile expanded to the first floral whorl, explaining the presence of petaloid sepals [19–21]. In addition, the orchid *AP3/DEF* lineage of the class B genes underwent two subsequent duplication events that have played an important role in the evolutionary origin of the current structure of the orchid flower described by the “orchid code” and the “homeotic orchid tepal” (HOT) models [22–24]. The more recent “P-code” model integrates the function of class B and *AGL6* MADS-box genes to explain the formation of the orchid perianth, particularly the lip [25].

**Fig 1:**
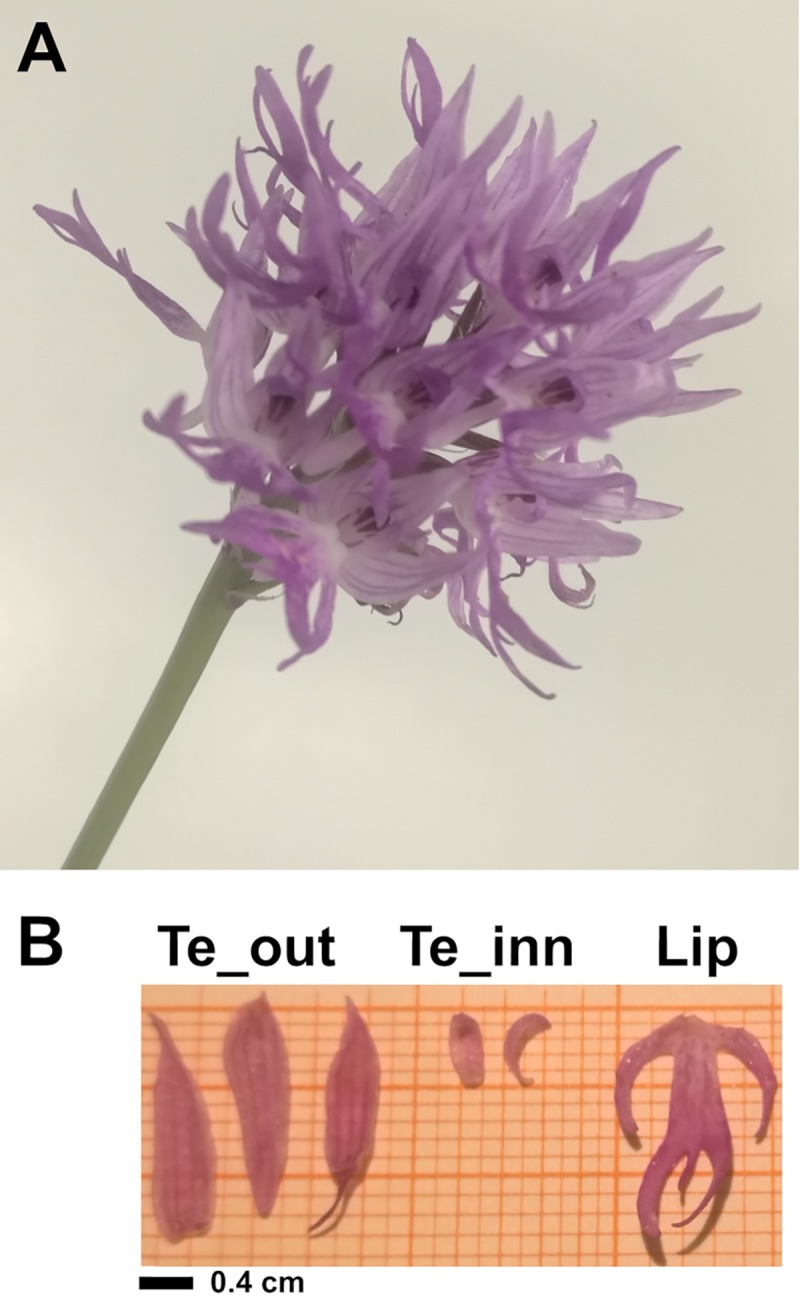
*Orchis italica*. **A**, inflorescence of *O*. *italica*; **B**, dissected outer and lateral inner tepals (Te_out and Te_inn, respectively) and lip of *O*. *italica*.

*Orchis italica* is an orchid species belonging to the subfamily Orchidoideae. It is one of the most widespread Mediterranean orchids with a white-purple cluster inflorescence and a lip with flaps that assume an anthropomorphic form (Fig 1). Previous studies have analyzed the structure, expression and evolution of some genes of *O*. *italica* involved in flower development. In particular, the floral meristem identity gene *LFY* [26], the AP2/ERF gene *AP2* (class A) [27] and the MADS-box genes *PI/GLO*, *AP3/DEF* (class B), *AG* (class C), *STK* (class D) and *AGL6* [20, 21, 27–33] have been assessed. However, the other MADS-box genes of *O*. *italica* have not yet been studied in contrast to other orchid species belonging to different subfamilies, mainly Epidendroideae [34–39]. Thus, the aim of this work was to identify the MADS-box genes expressed in the inflorescence of *O*. *italica*, analyze their expression in floral tissues, evaluate their evolutionary rate and compare the results with those reported in other orchids.

## Materials and methods

### Plant material

The *Orchis italica* plants used in this study (Fig 1A) are grown in the greenhouse at the Department of Biology, University of Naples Federico II. After anthesis, single florets were collected and outer tepals, lateral inner tepals, lips (Fig 1B), column and ovary (before pollination) were dissected and stored in RNALater^TM^ (Ambion) at −80°C for subsequent RNA extraction. Tissues from 10 flowers of two different plants were separately pooled and used for expression analysis.

### Isolation of the MADS-box transcripts of *O*. *italica*

To identify the transcripts encoding for the MADS-box proteins expressed in the floral tissues, a TBLASTN search was conducted against the inflorescence transcriptome of *O*. *italica* [40] using the MADS domain of the proteins PI/GLO, AP3/DEF, AG and STK of *O*. *italica* as queries [20, 21, 30–32]. The transcripts with significant hits were virtually translated to exclude those missing the domains downstream the MADS and/or containing premature stop codons. The MADS-box coding sequences (CDSs) identified in the genome of the orchids *Phalaenopsis equestris* (Epidendroideae) [36], *P*. *aphrodite* [37] and *Apostasia shenzenica* (Apostasioideae) [35] were downloaded from GenBank and Orchidstra2 (http://orchidstra2.abrc.sinica.edu.tw/orchidstra2/pagenome.php) along with MADS-box protein and nucleotide sequences of other plant species (S1 Table).

To annotate the selected transcripts of *O*. *italica*, BLASTN and BLASTX searches were conducted using the transcripts as queries against the Viridiplantae nucleotide and protein database, respectively.

The nucleotide sequence of all the transcripts of *O*. *italica* analyzed in this study was verified by PCR amplification and sequencing using the cDNA of inflorescence of *O*. *italica* as template [33]. The sequences of the primers used are reported in S2 Table.

### Phylogenetic and evolutionary analysis

The amino acid alignment of the MADS-box proteins of *O*. *italica*, *Phalaenopsis* and *A*. *shenzenica* was performed using MAFFT [41] and manually adjusted. The maximum likelihood (ML) tree was constructed using PhyML [42], computing the branch support values with approximate likelihood ratio tests.

The amino acid sequences of the MADS-box proteins of *O*. *italica* belonging to the AP1/FUL, SEP and AGL6 subfamilies were separately aligned to the homolog proteins of different orchids and other plant species (S1 Table), and phylogenetic trees were generated as described above. Protein-based nucleotide alignment of the orchid AP1/FUL, SEP and AGL6 subfamilies was produced using PAL2NAL [43].

The evolutionary rates of the AP1/FUL, SEP and AGL6 CDSs of *O*. *italica* and other orchids were analyzed using CODEML program from PAML v.4.9 [44]. Branch and branch-sites evolutionary models were tested, and the ratio between nonsynonymous and synonymous substitution rates (ω) was measured. The branch models assume one (one-ratio) or different (two- and three-ratios) ω values among the tree branches. The branch-site models test for positive selection on individual codons in specific branches of the tree and are compared with the respective null models that assume purifying or nearly neutral selection in the same branches. To establish which model best fits the data, a likelihood ratio test was applied comparing the alternative branch and branch-site models (allowing different ω or positive selection, respectively) with the corresponding null models.

### miRNA putative target analysis

The psRNATarget web server [45] was used to predict the presence of miRNA putative target sites on the MADS-box transcripts of *O*. *italica*. The analysis (maximum expectation value 0.0) was conducted using the miRNAs of *O*. *italica* [30] as small RNA library and all the transcripts of *O*. *italica* analyzed in this study as target library.

### Expression analysis

Total RNA was extracted from outer tepals (Te_out), inner lateral tepals (Te_inn), labellum (Lip), column (Co), and ovary (Ov) of *O*. *italica* after anthesis using the PureLink RNA Mini Kit (Invitrogen). Total RNA was also extracted from leaf tissue (Le). To evaluate the expression profile of the MADS-box transcripts identified in *O*. *italica*, reverse transcription and real time PCR reactions were conducted as previously reported [33, 46] using the specific primer pairs listed in S2 Table. For each transcript and tissue, the mean relative expression and standard error (SE) were calculated among three technical and two biological replicates. ANOVA followed by Tukey’s post hoc test was performed to assess the statistical significance of the mean relative expression differences among the tissues.

## Results and discussion

**Fig 2:**
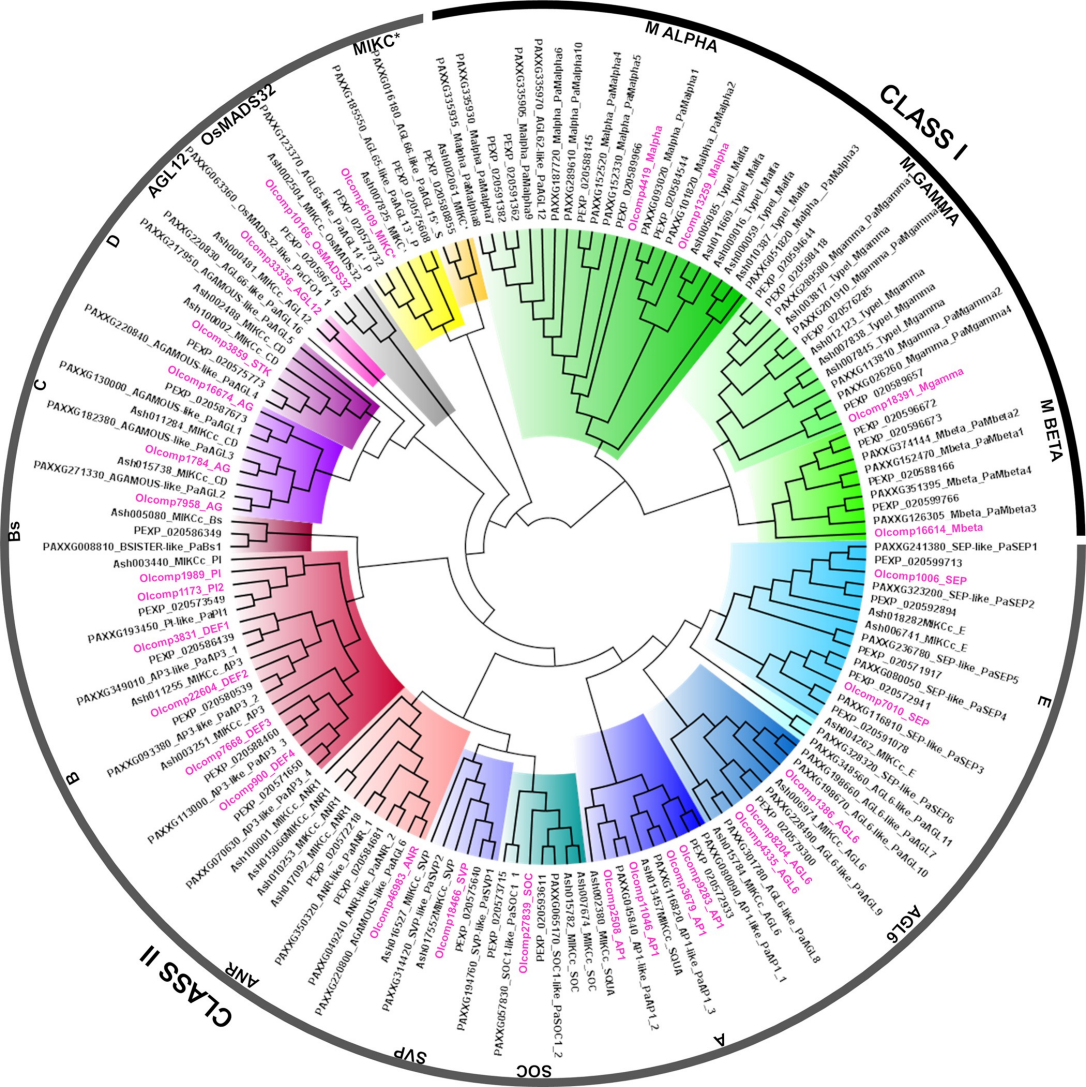
ML tree of the orchid MADS-box proteins. The different classes and groups of MADS-box proteins of *O*. *italica*, *P*. *aphrodite*, *P*. *equestris* and *A*. *shenzenica* are highlighted with different colors. The accessions of *O*. *italica* are noted in pink.

**Fig 3:**
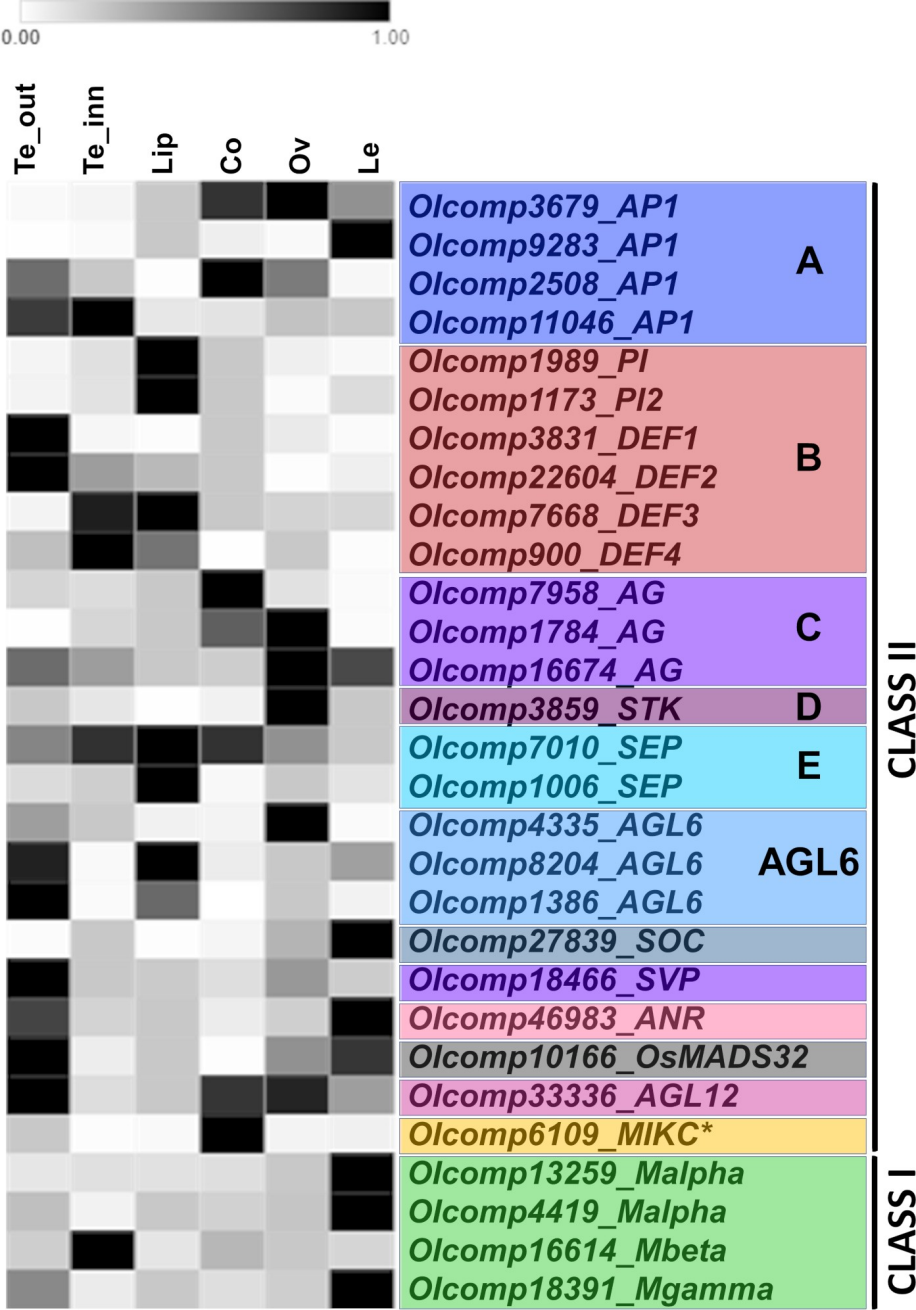
Heatmap of the relative gene expression of the MADS-box genes in the floral tissues of *O*. *italica*. The scale for each gene is independent of each other and set to 1 as the highest value. Te_out, outer tepals; Te_inn, lateral inner tepals; Co, column; Ov, ovary; Le, leaf.

The inflorescence transcriptome of *O*. *italica* [40] includes twenty-nine transcripts encoding for MADS-box proteins. This number is lower than that of the MADS-box genes present in the genome of *P*. *equestris* (51), *D*. *catenatum* (63) and *A*. *shenzenica* (36) [35, 36, 38] because it does not include the transcripts specifically expressed in not-floral tissues (e.g., leaf, root, stem, etc.). BLAST analysis and phylogenetic reconstruction (Fig 2) demonstrated that the MADS-box genes expressed in the inflorescence of *O*. *italica* belong to both class I and II. Excluding three class I and three class II genes (*SOC*, *ANR* and one class A), they show floral specific expression or are expressed in floral tissues at levels higher than in leaves (Fig 3).

### Phylogenetic and expression analysis of Class I MADS-box in *O*. *italica*

The four class I proteins of *O*. *italica* contain a conserved MADS domain (S1A Fig). Among them, two belong to the Mα type, one to Mγ and one to Mβ (Fig 2), confirming the presence of Mβ genes in orchids recently reported in *P*. *aphrodite* [37] and previously proposed to be absent from the orchid genomes [35]. The class I MADS-box transcripts of *O*. *italica* are poorly expressed in all the floral tissues (S2 Fig), as expected based on their role in embryo and endosperm maturation [5] and consistent with the low expression in the floral organs of most of the class I MADS-box genes in the orchid *A*. *shenzenica* [35]. Despite at low levels, all the class I genes identified in *O*. *italica* (especially the two Mα) are expressed in the column (at whose top are located the pollen grains) (Fig 3, S3 Fig). This pattern supports their involvement in the development of the orchid pollinia, which is consistent with the results obtained in *P*. *aphrodite*, where two Mα and three Mγ genes are specifically expressed in the pollen [37]. Their different expression in tepals, lip, ovary and leaf suggests a possible role also in other orchid floral and vegetative organs.

### Phylogenetic and expression analysis of Class II MADS-box in *O*. italica MIKC*

The only MIKC* transcript found in the transcriptome of *O*. *italica* belongs to the P-subclade (Fig 2). This transcript encodes for a MIKC protein exhibiting a K domain longer than that of the MIKC^C^ transcription factors (S1B Fig) as reported for the MIKC* proteins [10]. This MIKC* gene is expressed at high levels in the column (Fig 3, S3 Fig), which is consistent with the conserved function of the MIKC* genes in gametophyte development [10, 47], as hypothesized for the orchid species *Erycina pusilla* [34] and *P*. *aphrodite* [37].

### SOC, SVP, ANR1, AGL12 and OsMADS32

Among the twenty-four MIKC^C^ transcripts identified in the inflorescence transcriptome of *O*. *italica*, two are homologs of MADS-box genes involved in the regulation of flowering (*SOC1* and *SVP*). Their expression in the floral tissues of *O*. *italica* is weak (S2 and S4 Figs), which is consistent with the expression profile detected in the flowers of the orchids *Dendrobium* [48, 49] and *Erycina pusilla* [34] and other plant species [50–52]. Two other transcripts only weakly expressed in the inflorescence of *O*. *italica* are homologs of MADS-box genes involved in root development (*AGL12* and *ANR1*) [53, 54]. The *AGL12* gene is present in the genome of terrestrial orchids (e.g., *Apostasia*) and seems to be absent in epiphytic orchids [35]. Its presence in *O*. *italica*, a terrestrial orchid species, supports its role in the formation of true roots typical of terrestrial plant species. Another MIKC^C^ transcript expressed at low levels in the floral tissues of *O*. *italica* is a homolog of *OsMADS32* (also named *CFO1*), a monocot-specific MADS-box gene that regulates flower organ identity [55, 56]. In rice and wheat, this gene is expressed in the early stages of the inflorescence and late seed development [50, 57], which could explain the low expression detected in the mature floral tissues of *O*. *italica*.

The MIKC^C^ transcripts most expressed in the floral tissues of *O*. *italica* belong to the A-E classes of MADS-box genes involved in the ABCDE model of flower development (S2 Fig).

### PI/GLO and AP3/DEF

Six transcript homologs of the class B genes, including two *PI/GLO* and four *AP3/DEF*, have been previously analyzed in *O*. *italica* [20, 21, 28–31]. Their expression (in particular of the *AP3/DEF* genes) is not restricted to the second and third floral whorls. Indeed, their expression is extended to the outer tepals (first whorl) (Fig 3), as described for orchids and other species exhibiting petaloid tepals in the first floral whorl [58]. In addition, the orchid *AP3/DEF* genes have been extensively studied from the evolutionary perspective and are considered the main genes responsible of the diversification of the orchid perianth, as described in the “orchid code” and HOT models [22–24].

### AG and STK

Also class C (*AG*) and D (*STK*) MADS box genes of *O*. *italica* have been previously analyzed [32]. However, in the present work, two additional transcripts belonging to class C have been identified (Fig 2). Both these transcripts encode for proteins that contain AG-motifs I and II at the C-terminus (S1C Fig) and share 69% of amino acid residues. The protein encoded by the transcript OIcomp1784 exhibits 85% identity with the class C OitaAG (and 62% with the class D OitaSTK), whereas the protein encoded by the transcript OIcomp16674 exhibits 71% identity with OitaAG (and 52% with OitaSTK). A transcript similar to *OIcomp16674* (84% identity) is also present in the transcriptome of *Ophrys sphegodes*, an orchid belonging to the Orchidoideae subfamily, as *O*. *italica*. The expression of class C and D transcripts in *O*. *italica* is high in the column and ovary (Fig 3, S5 Fig), and this pattern is consistent with the expression of the C and D genes in other orchid species, as they are involved in development of the female reproductive structure [59–61]. The newly identified transcript *OIcomp16674* is also detectable in the other floral organs albeit at low levels (S5 Fig). The presence of three *AG* genes and one *STK* gene in *O*. *italica* is consistent with the copy number of class C and D MADS-box genes of *Erycina pusilla* [34, 39], as well as their expression pattern [39].

Excluding two *AGL6* transcripts [33], class A and E genes of *O*. *italica* have never been studied. In the inflorescence of *O*. *italica*, four class A, two class E, and three *AGL6* transcripts are expressed, increasing the number of *AGL6* transcripts previously reported in *O*. *italica*.

### AP1/FUL

**Fig 4:**
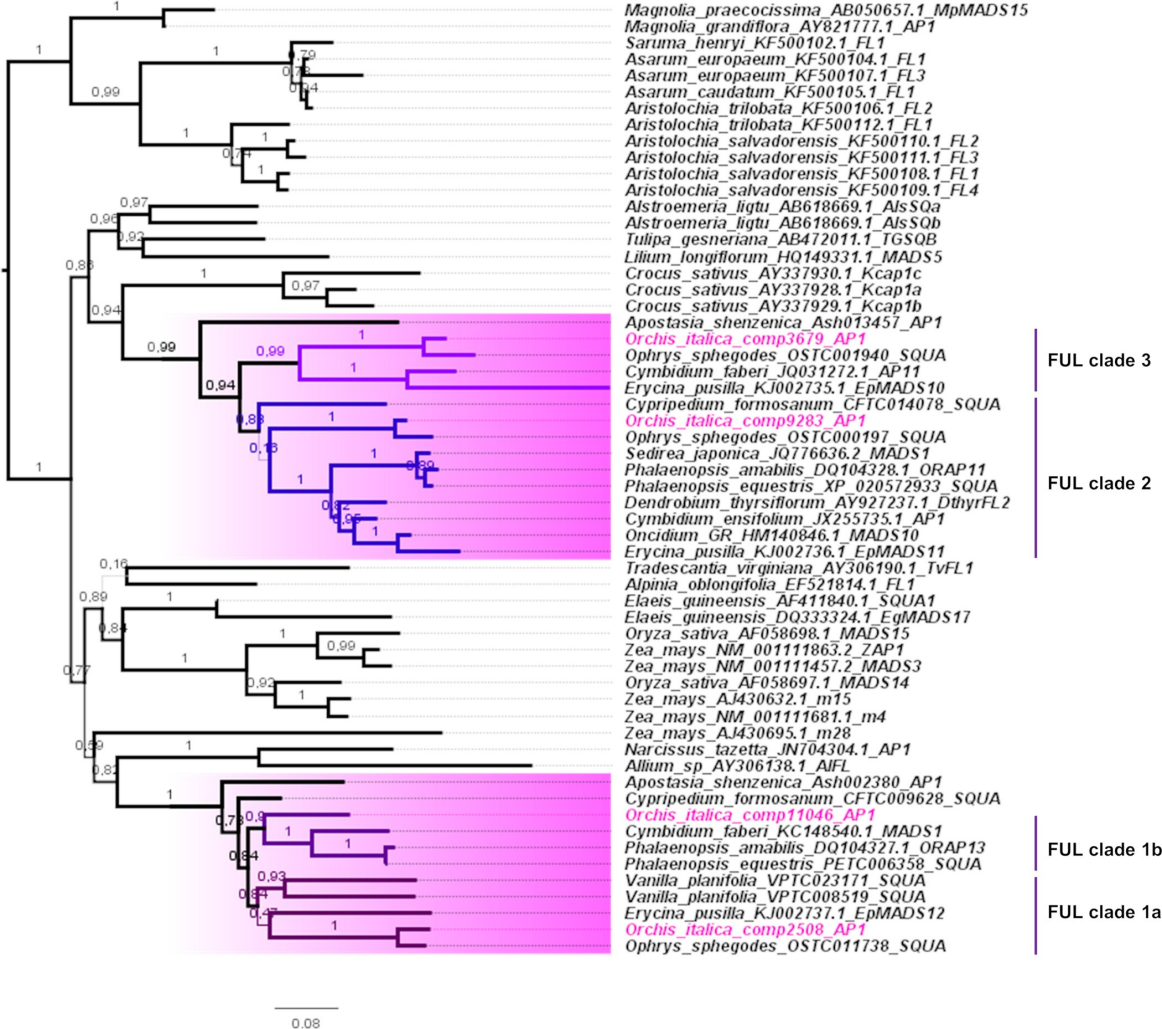
ML tree of the Class A MADS-box proteins. The orchid branches are highlighted in pink, and orchid FUL clades 1–3 are indicated. The accessions of *O*. *italica* are noted in pink. The numbers indicate the statistical support of the branches.

The four class A transcripts of *O*. *italica* belong to the two monocot FUL-like lineages previously identified [62] (Fig 4). Within each FUL-like lineage, orchid-specific duplications expanded the copy number of the *AP1/FUL* genes, defining four well supported orchid FUL clades (1a, 1b, 2 and 3). Three *AP1/FUL* transcripts of *O*. *italica* encode for proteins exhibiting the complete FUL-like motif LPPWML at the C-terminus (S1D Fig). The protein encoded by the transcript *OIcomp3679* lacks the FUL-like motif as observed in other AP1/FUL orchid proteins of species belonging to Epidendroideae [59] and Apostasioideae (S1D Fig). This finding suggests that the divergence of the C-terminus of some orchid AP1/FUL proteins is ancient given that Apostasioideae are the most basal orchid subfamily [2]. The expression of the *AP1/FUL* transcripts *OIcomp3679*, *9283* and *2508* is high in column and ovary, and the two latter transcripts are also detectable in the outer and inner tepals (Fig 3, S5 Fig). This expression profile is very similar to that of *EpMADS10*, *11* and *12* of *E*. *pusilla* [34, 39]. In contrast, expression of the *OIcomp11046* transcript is increased in outer and inner tepals compared with column and ovary (Fig 3, S5 Fig). Although evolutionary analysis did not reveal positive selection signals within the *AP1/FUL* coding sequences of orchids, relaxation of purifying selection is strongly supported (Table 1, S3 Table). In fact, the ω value of the orchid *AP1/FUL* genes (0.23) is significantly increased compared with orchid *SEP* (0.11) and *AGL6* (0.14) genes. This result together with a previous report of diversifying selection detected in the orchid *AP1/FUL* genes [39], the divergence of the C-terminus sequence of some members of this clade in orchids and the peculiar expression profile of *OIcomp11046* suggest a possible functional diversification after duplication within these orchid genes.

**Table 1:** Models used and parameters estimated under different conditions for the evolutionary analysis of the orchid class A, *AGL6* and class E MADS-box genes. The parameter estimates of the branch and branch-site models were obtained from the CODEML analysis. Fore, foreground branch; back, background branch; param, parameters. The results of the LRT are reported in S3 Table.

Branch models						
	Fore_branch	ω_0	ω_1	ω_2	lnL	#param
**one-ratio**	//	0.15407	//	//	-25962.03	165
**two-ratios**	A	0.11796	0.23284	//	-25899.97	166
**two-ratios**	AGL6	0.16162	0.13553	//	-25958.58	166
**two-ratios**	E	0.18707	0.10409	//	-25919.46	166
**three-ratios**	A *(1)* AGL6 *(2)*	0.10520	0.23278	0.13537	-25894.84	167
**Branch-site models**						
**Site class**	**0**	**1**	**2a**	**2b**	**lnL**	**#param**
**% sites**	60.245	6.789	29.628	3.339		
**alternative_A_back_ω**	0.1093	1	0.1093	1	-25506.04	168
**alternative_A_fore_ω**	0.1093	1	1	1		
**null_A_back_ω**	0.1093	1	0.1093	1	-25506.04	167
**null_A_fore_ω**	0.1093	1	1	1		
**% sites**	73.021	19.147	6.205	1.627		
**alternative_AGL6_back_ω**	0.12294	1	0.12294	1	-25646.91	168
**alternative_AGL6_fore_ω**	0.12294	1	1	1		
**null_AGL6_back_ω**	0.12294	1	0.12294	1	-25646.91	167
**null_AGL6_fore_ω**	0.12294	1	1	1		
**% sites**	75.533	18.282	4.98	1.205		
**alternative_E_back_ω**	0.12467	1	0.12467	1	-25643.73	168
**alternative_E_fore_ω**	0.12467	1	1	1		
**null_E_back_ω**	0.12467	1	0.12467	1	-25643.73	167
**null_E_fore_ω**	0.12467	1	1	1		

### SEP

**Fig 5:**
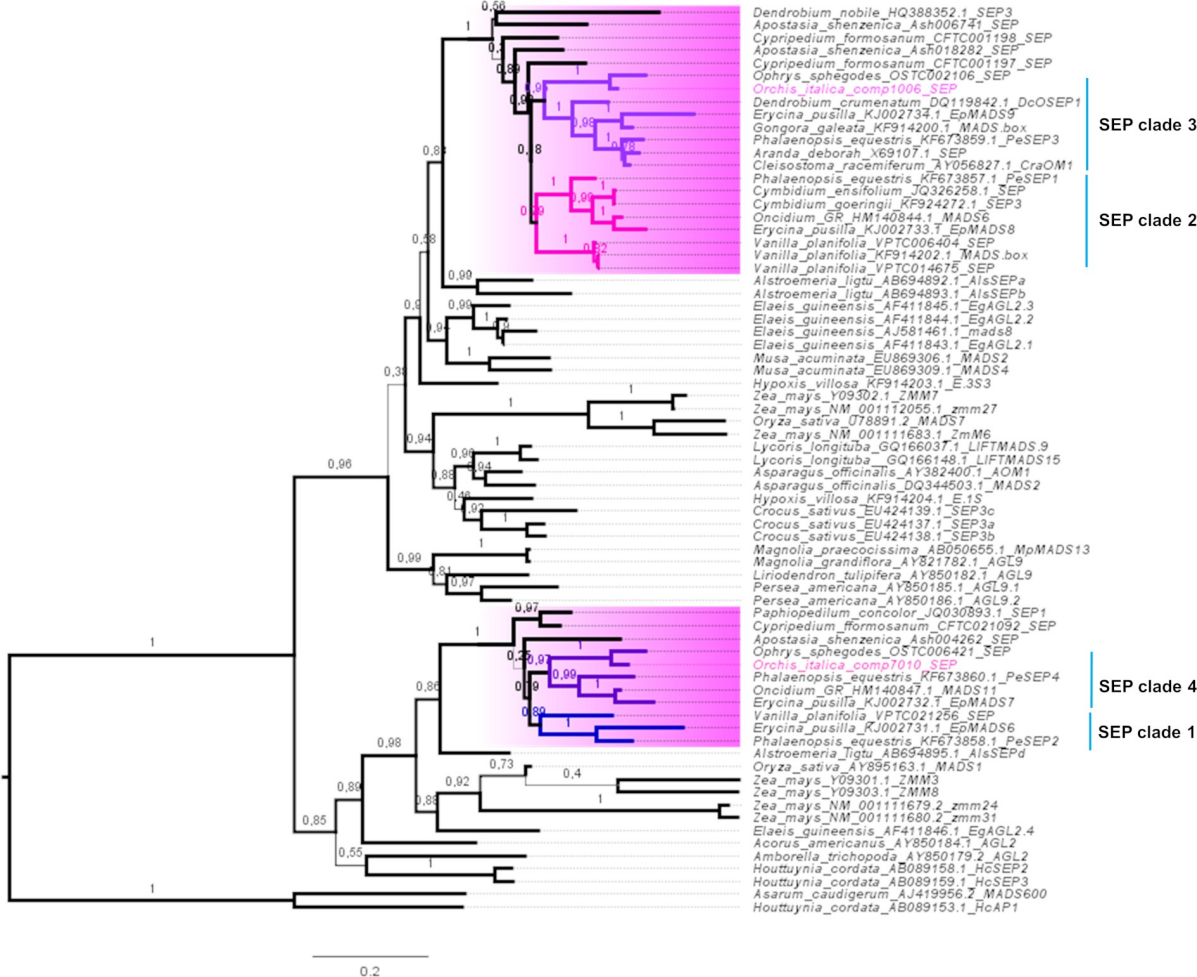
ML tree of the class E MADS-box proteins. The orchid branches are highlighted in pink, and the orchid SEP clades 1–4 are indicated. The accessions of *O*. *italica* are noted in pink. The numbers indicate the statistical support of the branches.

Two transcripts expressed in the inflorescence of *O*. *italica* encode for SEP proteins that harbor both the SEP I and II motifs (S1E Fig) and share 60% identity. In orchids, four clades of *SEP* genes have been reported that were generated from orchid-specific duplications within monocot SEP clades [34, 39, 59]. One SEP sequence of *O*. *italica* belongs to the clade that includes EpMADS9 and PeSEP3 (SEP clade 3), whereas the other SEP sequence is grouped with EpMADS7 and PeSEP4 (SEP clade 4) (Fig 5). BLAST search for *SEP* transcripts in the transcriptome of *Ophrys sphegodes* (Orchidoideae) [63] produced two significant hits, both exhibiting high sequence identity with the *SEP* transcripts identified in *O*. *italica*. In addition, a recent study identified two *SEP* transcripts expressed in the floral buds of *Habenaria radiata* (Orchidoideae) [64]. This evidence suggests that only two *SEP* genes are present or that only two *SEP* genes are expressed in the floral tissues in the subfamily Orchidoideae. Unfortunately, genome data are not currently available for orchid species belonging to Orchidoideae, rendering it difficult to discriminate between the two hypotheses. In both cases, the expression of *SEP* genes in *O*. *italica* is detectable in all floral organs (Fig 3, S6 Fig), and *OIcomp1006* exhibits significantly increased expression in lip. This pattern is similar to that reported in wild type *H*. *radiata* [64] and other orchid species, such as the Epidendroideae *P*. *equestris* [59] and *E*. *pusilla* [34, 39], and this finding is consistent with the expression pattern in all the floral whorls of class E genes involved in the formation of all the organs of the flower.

### AGL6

**Fig 6:**
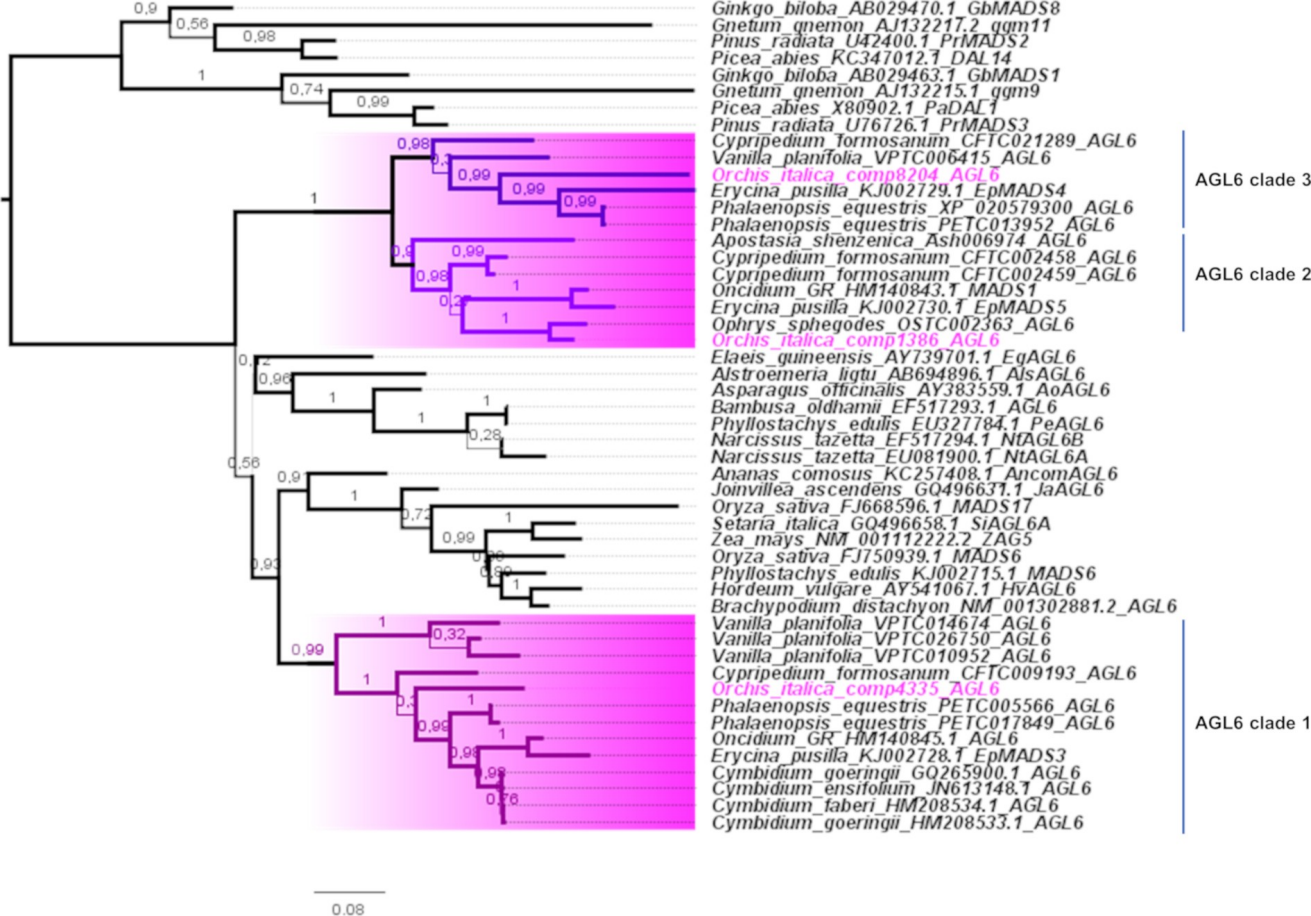
ML tree of the AGL6 MADS-box proteins. The orchid branches are highlighted in pink, and the orchid AGL6 clades 1–3 are indicated. The accessions of *O*. *italica* are noted in pink. The numbers indicate the statistical support of the branches.

**Fig 7:**
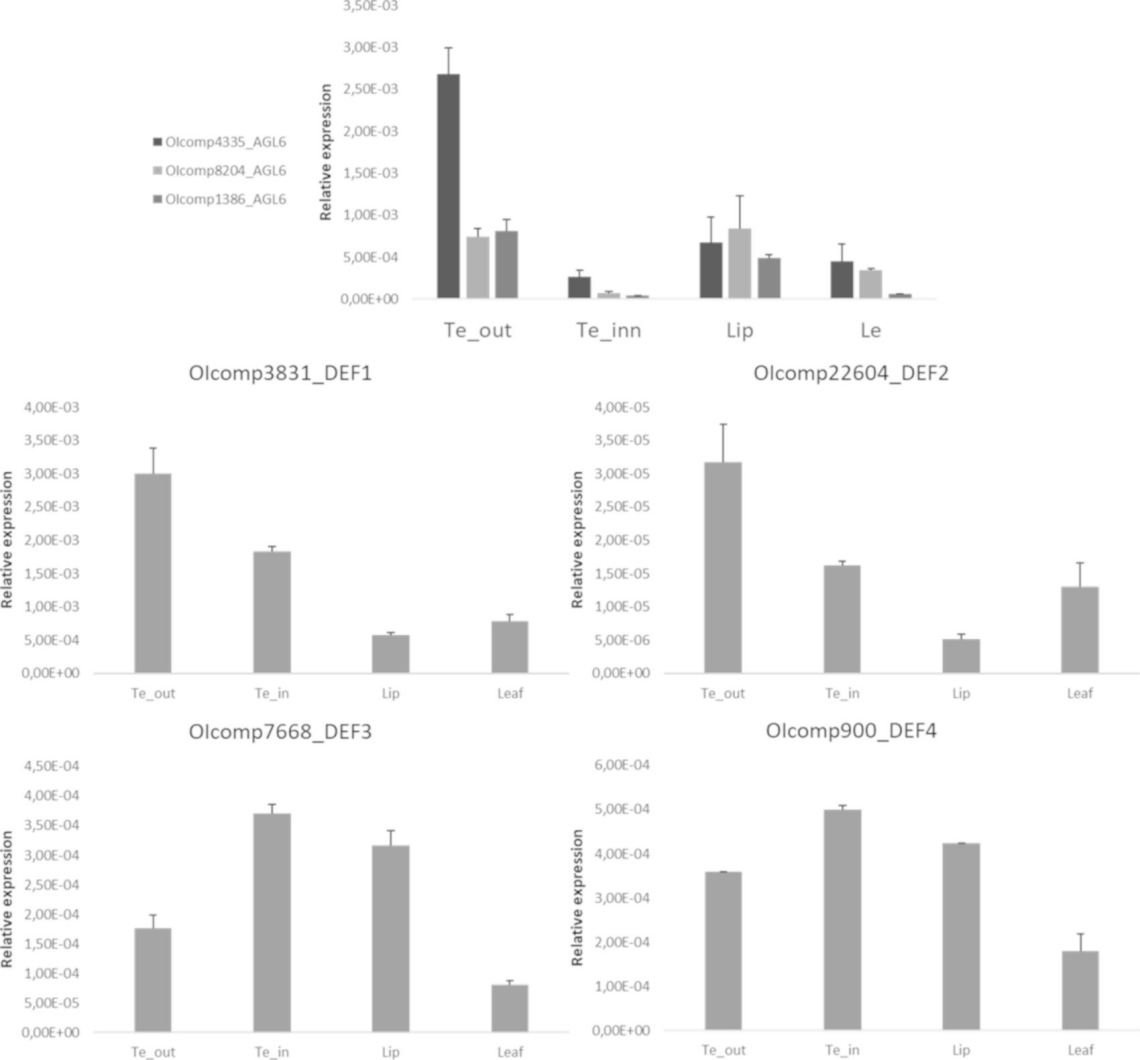
Expression pattern of the *AGL6* and *AP3/DEF* MADS-box genes in the perianth of *O*. *italica*. Each column of the *AGL6* genes indicates the relative expression of 10 floral organs in two cDNA pools (10 floral organs from two different plants), both of which are amplified in triplicate. The error bars represent the standard error of the mean. Te_out, outer tepals; Te_inn, inner tepals; Le, leaf.

The three *AGL6* transcripts expressed in the inflorescence of *O*. *italica* encode for proteins harboring both conserved AGL6-motifs I and II at the C-terminus (S1F Fig). In orchids, two main AGL6 clades have been reported: one includes other monocot species and one includes only orchid species [25, 37]. The three *AGL6* transcripts found in *O*. *italica* belong to these two clades, and the tree topology clearly show a duplication within the orchid-specific AGL6 clade that gave rise to two different, well supported paralog groups: AGL6 clades 2 and 3 (Fig 6). Both the clades include basal orchid species (Apostasioideae, Cypripedioideae or Vanilloideae) in addition to Epidendroideae and Orchidoideae, demonstrating the ancient origin of these paralog groups. The newly identified *AGL6* transcript *OIcomp8204* is the paralog of *OIcomp1386* [33]. These genes have an overlapping expression profile (Fig 3, S6 Fig), suggesting a redundant functional role. They are mostly expressed in lip and outer tepals, whereas *OIcomp4335* exhibits high expression in the ovary and outer tepals and lower expression in lip. However, comparing the expression of these three *AGL6* genes, similar levels are detected in lips, whereas *OIcomp4335* expression is significantly increased in tepals (Fig 7). Orchid *AGL6* genes together with *AP3/DEF* genes drive the formation of the orchid perianth. In particular, the “P-code” model proposes the formation of SP and L protein complexes that include combinations of different AGL6 and AP3/DEF proteins. The SP complex determines the formation of the tepals, and the L complex determines the formation of lip [25]. Given that the *AGL6* transcripts of *O*. *italica* are expressed at similar levels in lip, whereas the four *AP3/DEF* genes exhibit different expression levels (Fig 7) [30], it is possible to hypothesize that the modulation of the expression level of the genes encoding for the different AP3/DEF proteins is responsible for the formation of the L or SP complex within the specific floral whorl.

The the presence of putative miRNA target sites on the twenty-nine MADS-box transcripts of *O*. *italica* identified in this study was predicted using the psRNATarget web server with a stringent parameter setting (maximum expectation 0.0). The analysis gave positive results only for the transcripts *DEF2* and *DEF4*, known targets of the *O*. *italica* miRNAs homologs of miR5179 [30]. The other MADS-box transcripts do not have any putative target site, showing that they are not regulated by miRNAs.

## Conclusions

Current advances in transcriptome and genome sequencing are highlighting the molecular programs that underlie floral evolution and development of non-model species. For many species belonging to basal angiosperms, magnoliids and basal eudicots, the “fading borders model” proposes a gradient of the expression levels of floral homeotic genes to explain flower development [65]. In particular, low expression levels at the boundary of the domain of a given MADS-box gene overlap with the expression of a different homeotic gene in the adjacent domain. The analysis of all the MADS-box genes expressed in the inflorescence of *O*. *italica* demonstrate that they follow a “fading borders” scheme and that their expression is generally conserved among orchids of different subfamilies. Given that class I and MIKC* genes are understudied compared with MIKC^C^ genes, it will be interesting to focus forthcoming studies on these classes of MADS-box genes in orchids to understand their developmental role and evolution.

## Supporting information

S1 Fig Amino acid motifs of the orchid MADS-box proteins. The sequences of selected orchid species are grouped according to the MADS-box type. **A**, class I MADS domain; **B**, I domain and part of the K domain of the MIKC* proteins; **C-F**, C-terminus of AG/STK, AP1/FUL, SEP and AGL6 proteins, respectively. The sequences of **A** and **B** are also aligned with the corresponding region of one DEF protein of *O*. *italica*. The conserved motifs of each clade of MADS-box proteins are indicated with a black line. The accessions of *O*. *italica* are noted in bold.
(TIF)

S2 Fig Relative expression of the MADS-box genes expressed in the inflorescence of *O*. *italica*. Each column of the MADS-box genes indicates the relative expression of 10 floral organs in two cDNA pools (10 floral organs from two different plants), both of which are amplified in triplicate. The error bars represent the standard error of the mean. Te_out, outer tepals; Te_inn, inner tepals; Co, column; Ov, ovary.
(TIF)

S3 Fig Relative expression of the class I and MIKC* MADS-box genes in floral tissues of *O*. *italica*. Each column of the class I and MIKC* genes represents the relative expression of 10 floral organs in two cDNA pools (10 floral organs from two different plants), both of which are amplified in triplicate. The error bars represent the standard error of the mean. Te_out, outer tepals; Te_inn, inner tepals; Co, column; Ov, ovary.
(TIF)

S4 Fig Relative expression of the *SOC*, *SVP*, *ANR1*, *AGL12* and *OsMADS32* genes in floral tissues of *O*. *italica*. Each column of the *SOC*, *SVP*, *ANR1*, *AGL12* and *OsMADS32* genes represents the relative expression of 10 floral organs in two cDNA pools (10 floral organs from two different plants), both of which are amplified in triplicate. The error bars represent the standard error of the mean. Te_out, outer tepals; Te_inn, inner tepals; Co, column; Ov, ovary.
(TIF)

S5 Fig Relative expression of the *AG* and class A MADS-box genes in floral tissues of *O*. *italica*. Each column of the class C *AG* and class A *AP1/FUL* genes indicates the relative expression of 10 floral organs in two cDNA pools (10 floral organs from two different plants), both of which are amplified in triplicate. The error bars represent the standard error of the mean. Te_out, outer tepals; Te_inn, inner tepals; Co, column; Ov, ovary.
(TIF)

S6 Fig Relative expression of the *AGL6* and class E MADS-box genes in floral tissues of *O*. *italica*. Each column of the *AGL6* and the class E *SEP* genes shows the relative expression of 10 floral organs in two cDNA pools (10 floral organs from two different plants), both of which are amplified in triplicate. The error bars represent the standard error of the mean. Te_out, outer tepals; Te_inn, inner tepals; Co, column; Ov, ovary.
(TIF)

S1 Table List of sequences used in the alignments, phylogenetic and evolutionary analyses. (XLSX)

S2 Table List of the primers used in the present work. (XLSX)

S3 Table Likelihood ratio statistics for the comparison of the evolutionary models of the orchid class A, *AGL6* and class E coding regions. df, degrees of freedom; ns, not significant.
(XLSX)
