# Supplementary figures and images for: The MADS-box genes expressed in the inflorescence of Orchis italica (Orchidaceae)
Source: PLoS One. 2019 Mar 1;14(3):e0213185. doi: 10.1371/journal.pone.0213185 (PMC6396907; doi:10.1371/journal.pone.0213185)

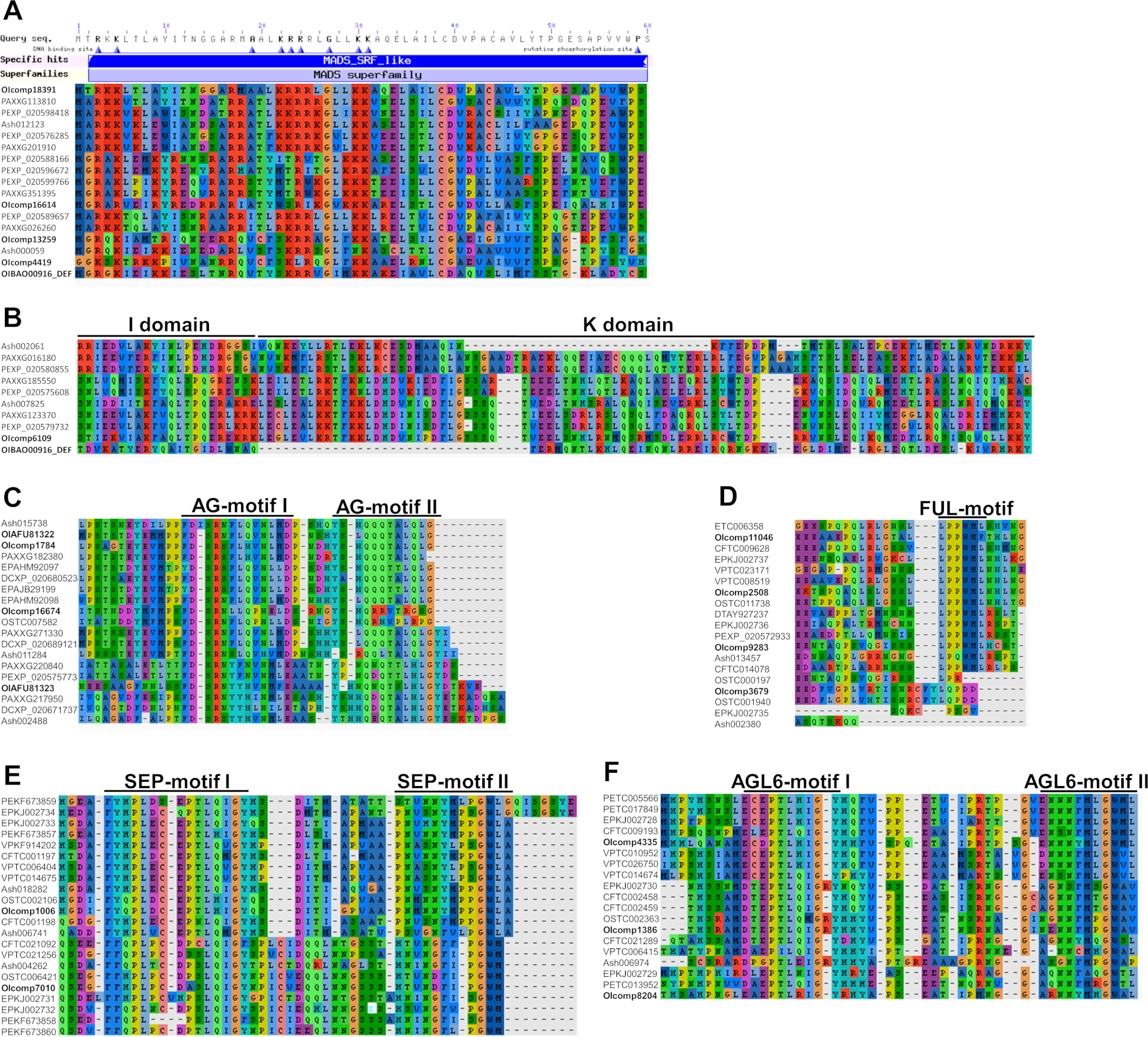

Supplement: S1 Fig — The sequences of selected orchid species are grouped according to the MADS-box type. A, class I MADS domain; B, I domain and part of the K domain of the MIKC* proteins; C-F, C-terminus of AG/STK, AP1/FUL, SEP and AGL6 proteins, respectively. The sequences of A and B are also aligned with the corresponding region of one DEF protein of O. italica. The conserved motifs of each clade of MADS-box proteins are indicated with a black line. The accessions of O. italica are noted in bold. (TIF) [file pone.0213185.s001.tif]

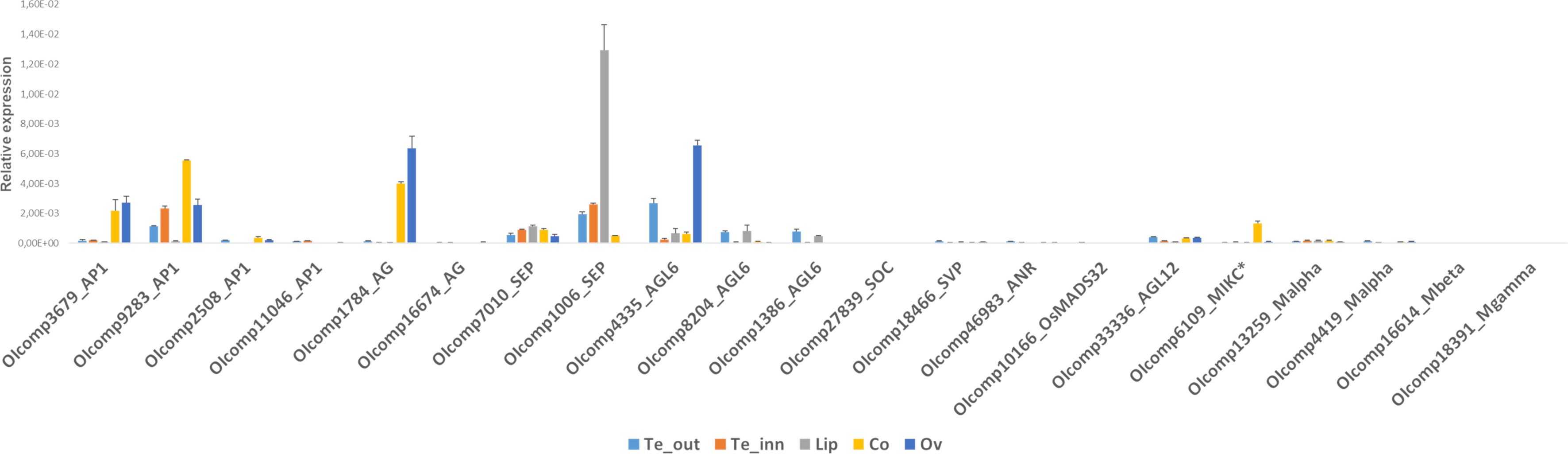

Supplement: S2 Fig — Each column of the MADS-box genes indicates the relative expression of 10 floral organs in two cDNA pools (10 floral organs from two different plants), both of which are amplified in triplicate. The error bars represent the standard error of the mean. Te_out, outer tepals; Te_inn, inner tepals; Co, column; Ov, ovary. (TIF) [file pone.0213185.s002.tif]

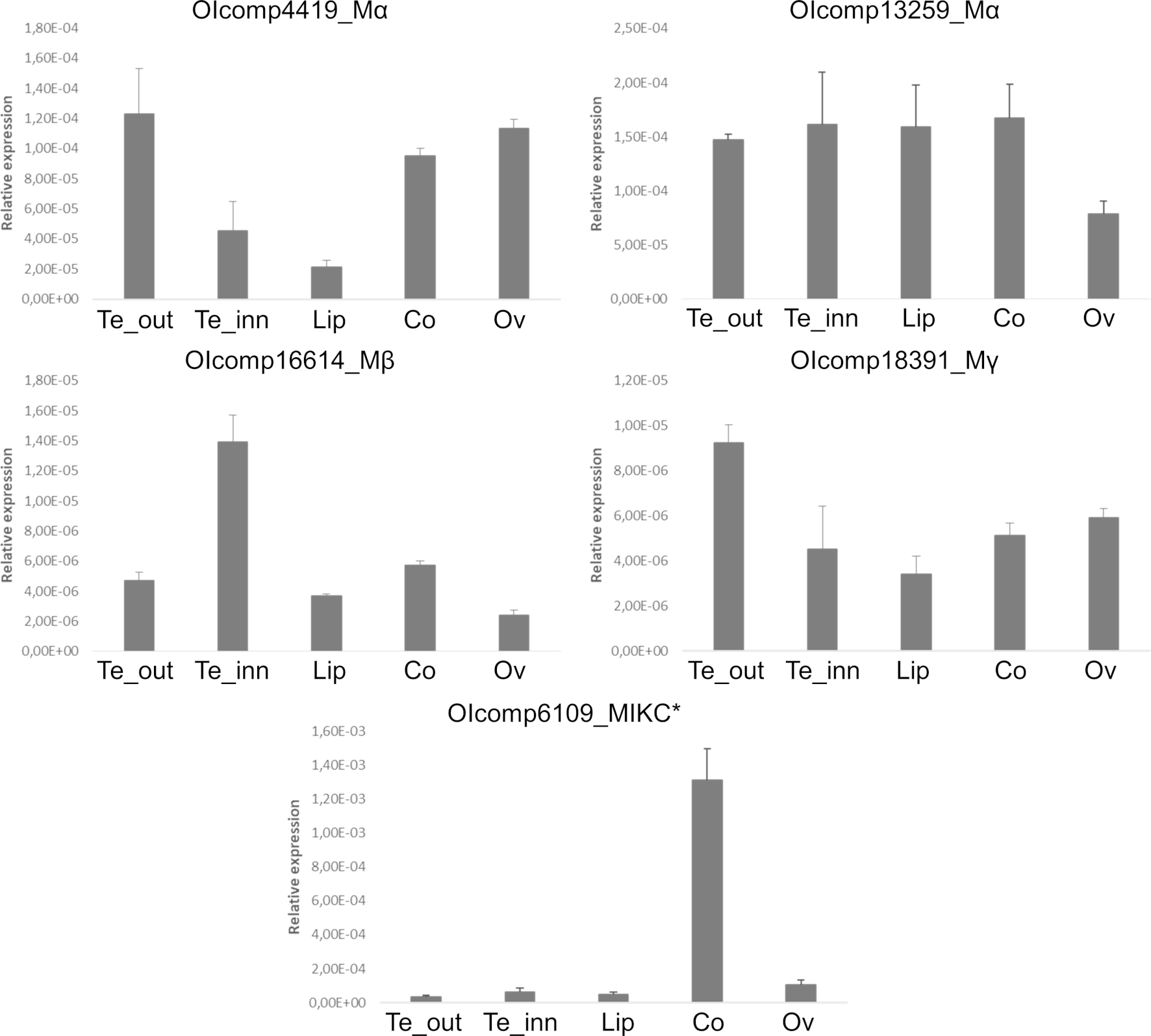

Supplement: S3 Fig — Each column of the class I and MIKC* genes represents the relative expression of 10 floral organs in two cDNA pools (10 floral organs from two different plants), both of which are amplified in triplicate. The error bars represent the standard error of the mean. Te_out, outer tepals; Te_inn, inner tepals; Co, column; Ov, ovary. (TIF) [file pone.0213185.s003.tif]

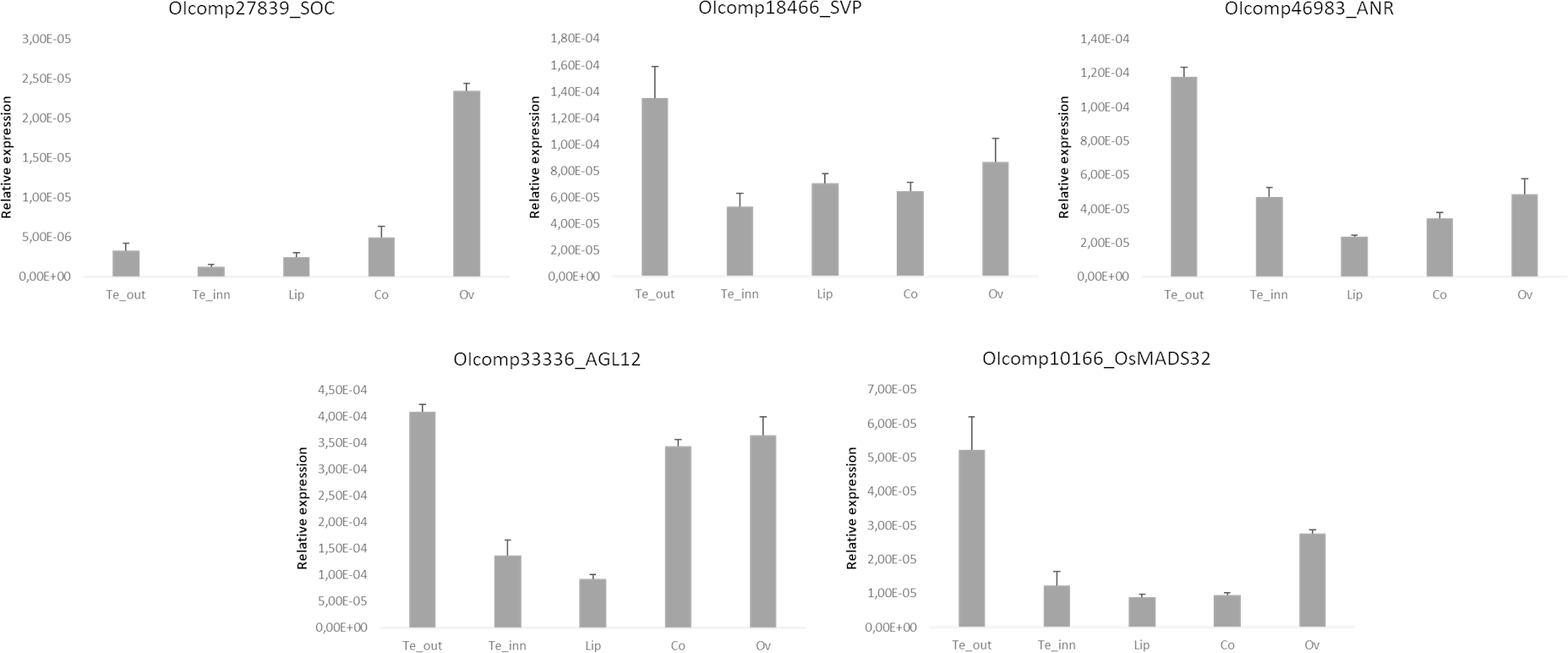

Supplement: S4 Fig — Each column of the SOC, SVP, ANR1, AGL12 and OsMADS32 genes represents the relative expression of 10 floral organs in two cDNA pools (10 floral organs from two different plants), both of which are amplified in triplicate. The error bars represent the standard error of the mean. Te_out, outer tepals; Te_inn, inner tepals; Co, column; Ov, ovary. (TIF) [file pone.0213185.s004.tif]

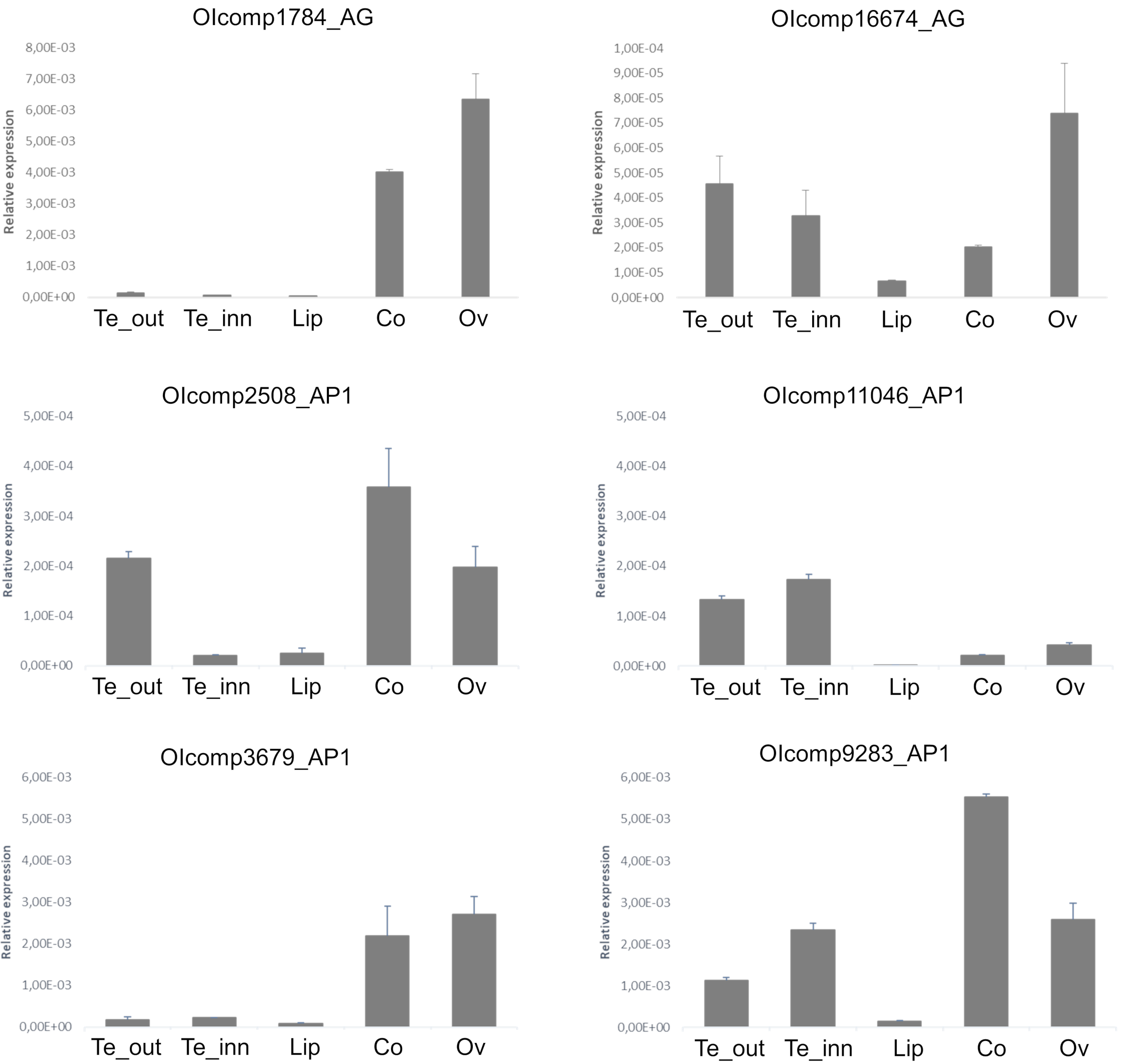

Supplement: S5 Fig — Each column of the class C AG and class A AP1/FUL genes indicates the relative expression of 10 floral organs in two cDNA pools (10 floral organs from two different plants), both of which are amplified in triplicate. The error bars represent the standard error of the mean. Te_out, outer tepals; Te_inn, inner tepals; Co, column; Ov, ovary. (TIF) [file pone.0213185.s005.tif]

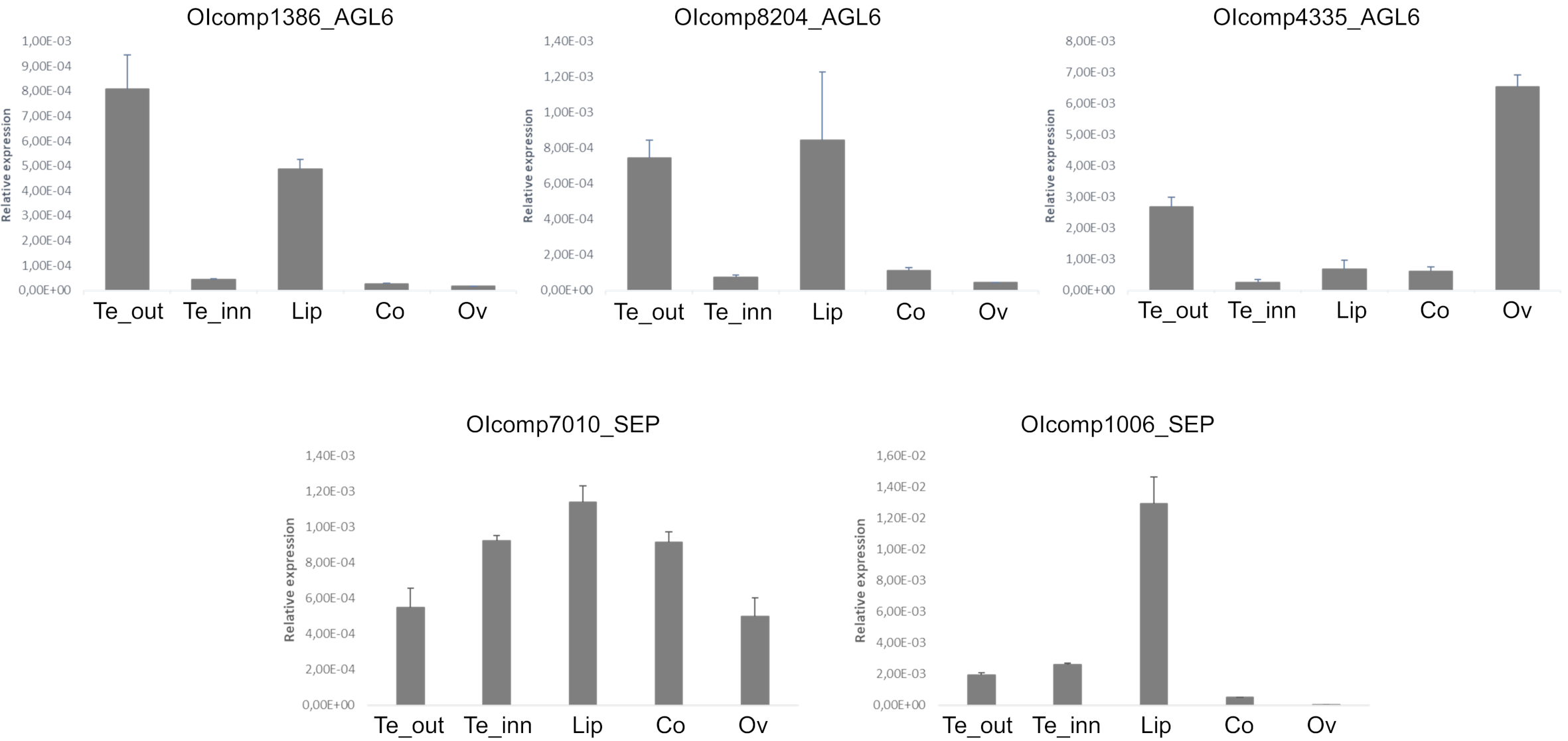

Supplement: S6 Fig — Each column of the AGL6 and the class E SEP genes shows the relative expression of 10 floral organs in two cDNA pools (10 floral organs from two different plants), both of which are amplified in triplicate. The error bars represent the standard error of the mean. Te_out, outer tepals; Te_inn, inner tepals; Co, column; Ov, ovary. (TIF) [file pone.0213185.s006.tif]
